# Cortical mechanisms of sensory learning and object recognition

**DOI:** 10.1098/rstb.2008.0271

**Published:** 2008-10-31

**Authors:** K.L. Hoffman, N.K. Logothetis

**Affiliations:** 1 Department of Psychology, York UniversityToronto, Ontario, Canada M3J 1P3; 2 Max Planck Institute for Biological Cybernetics80539 Munich, Germany; 3 Imaging Science and Biomedical Engineering, University of ManchesterManchester M13 9PL, UK

**Keywords:** face processing, amodal perception, spike timing, invariance

## Abstract

Learning about the world through our senses constrains our ability to recognise our surroundings. Experience shapes perception. What is the neural basis for object recognition and how are learning-induced changes in recognition manifested in neural populations? We consider first the location of neurons that appear to be critical for object recognition, before describing what is known about their function. Two complementary processes of object recognition are considered: discrimination among diagnostic object features and generalization across non-diagnostic features. Neural plasticity appears to underlie the development of discrimination and generalization for a given set of features, though tracking these changes directly over the course of learning has remained an elusive task.

## 1. Introduction

Perception does not occur as the tabula rasa. Even newborns come into the world with biases that point them along the path of learning about the faces and places surrounding them. One of the most constructive processes in perception is object recognition, since our three-dimensional understanding of the objects around us are known to us only via brief, often occluded, two-dimensional blips somewhere on our retina. The rest of the process is up to our brains, and will be based on a foundation of extensive visual experience. What is the nature of this constructive process? What parts of the brain are critical for object recognition and how does it enable learning about new objects and object categories?

In this article, we will consider first where and then how the brain learns to recognise objects. The ‘where’ description will involve neurons in the lateral and ventral temporal lobe neocortex, though other areas have also been implicated. The ‘how’ can be thought of as an interplay between discrimination, in which features are teased apart, and generalization or invariance, in which features are treated as the same. The former, discrimination, is the process by which perceptual learning is classically interpreted. Typically, these experiments take a relatively ‘elemental’ aspect of a visual stimulus, such as line orientation, and train to increasing levels of discriminability. But equally important to object learning is the development of generalization across irrelevant changes in features. For example, we maintain a remarkable ability to recognise objects across dramatic changes in appearance due to different lighting or angle of regard. We will consider the mechanisms that may underlie discrimination, or increased sensitivity to visual features, before tackling the more elusive issue that is the counterpart to sensitivity: generalization, or grouping across varying features.

One limitation that should be mentioned at the outset is that any description of the mechanisms of object learning will rely heavily on the neural coding of objects, with the assumption that this has been built up through experience. Nearly all studies on the neural basis of object learning and categorization monitor neural responses before and after learning, rather than tracking the process itself (but see Messinger *et al*. (2001) for one notable exception). Responses to novel stimuli (or features) are compared to those of trained stimuli (or features), and any difference in the neural code is attributed to learning. Prior biases between novel and trained stimuli, or use of innately specified or otherwise unique object categories, may render spurious results. This is still the predominant method, since it does not require recording from the same neurons over the hours, days or months of learning, making it accessible to most of the recording methods available. Adding to the technical challenges is the location in which object-selective responses are seen: areas at the lateral and ventral extreme in human and macaque brains, which are more difficult to access from the standard dorsal approach. Section 1*a* will describe in more detail the brain regions that are associated with object recognition.

### (a) The ventro-lateral temporal lobe encodes shapes and objects

Inferotemporal cortex (IT), lying along the lateral and ventral aspects of the temporal lobe, is the first visual area in which no retinotopic organization is seen. In contrast to most other visual areas in the brain, responses are largely position invariant, with receptive fields that cover major portions of our field of view, typically overlapping the fovea, crossing hemifields (adapted from Gross *et al*. 1972; Desimone & Gross 1979; Desimone *et al*. 1984). Stimuli that reliably drive cells in primary visual areas are far less effective in IT (Gross *et al*. 1972; Richmond *et al*. 1983; Pollen *et al*. 1984; Vogels & Orban 1994*a*); instead, faces and objects are commonly observed to drive cells in IT (Perrett *et al*. 1982; Rolls & Baylis 1986; Yamane *et al*. 1988; Hasselmo *et al*. 1989; Young & Yamane 1992). More recent imaging results are generally consistent with the location and selectivity of cells in superior temporal sulcus (STS)/IT (Logothetis *et al*. 1999; Tsao *et al*. 2003; Pinsk *et al*. 2005; Zangenehpour & Chaudhuri 2005; Hoffman *et al*. 2007). The importance of IT in processing shapes, objects and faces has been demonstrated through lesions or disconnections in IT (Mishkin 1954; Mishkin & Pribram 1954; Horel & Misantone 1976; Ungerleider & Mishkin 1982) and, for face discrimination, inactivation of STS, medial temporal gyrus or inferior temporal gyrus all produce impairments (Horel *et al*. 1987; Horel 1993). Conversely, stimulation within areas selective for faces elicits a bias towards face perception (Afraz *et al*. 2006). Given the importance of the code in IT for discriminating objects, we will focus on this region in our discussion on the neural basis of perceptual learning of objects. Because relatively few studies have explored the effects of learning in any one sub-region within the IT, we will consider in our discussion studies exploring the broadly defined territory around IT, including the upper bank of STS and perirhinal cortex (PRh).

## 2. Object recognition I: increased sensitivity

**Figure 1:**
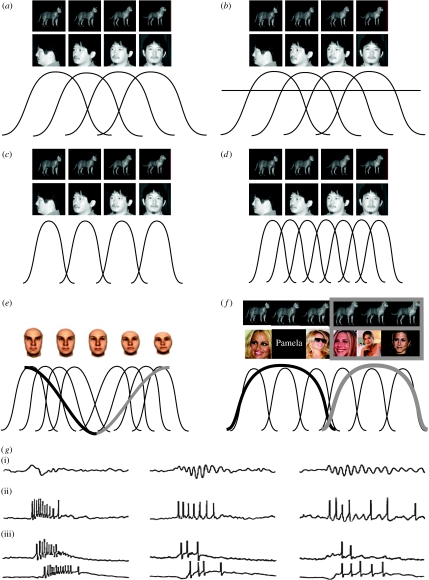
(*a*) Schematic of tuning curves for systematically varying face or object stimuli. Each black curve shown below reflects the relative change in firing rate for a given neuron that is elicited by the stimuli depicted above. The neuron leading to the far left curve would be said to ‘prefer’ the profile face view (or cat stimulus), but would also change activity for the adjacent image. (*b*) Cat and dog morphs taken from Freedman *et al*. (2003); face views taken from Eifuku *et al*. (2004). Selectivity can be increased by raising thresholds, as indicated by the horizontal black line. Here, instead of firing at an intermediate level for the adjacent images, the tuning curves indicate a near or below threshold activity level for all but the preferred stimulus. This decrease in the number of effective stimuli is also referred to in the text as a ‘sparsening’ of the code. (*c*) Sparsening shown ‘normalised’ to the threshold. The same tuning functions as in (*b*), but shown with respect to the new threshold. The narrowing of the tuning curve, indicating sparsening, is now clear. (*d*) Increased sensitivity to the varying stimulus parameter can be accomplished by a combination of the recruitment of neurons and a sparsening (i.e. sharpening) of their tuning functions. (*e*) Selective sharpening and recruitment restricted to the critical parameters is sometimes seen. For example, sharpening of tuning can be seen around trained orientations. In contrast to increased sensitivity to small differences, categorization effects are often seen as an invariance to small, irrelevant differences, but a preserved sensitivity to the across-category, or relevant, differences. The extreme right and left faces here represent two identities, with intermediate morphs in between, and the prototype morph in the middle (adapted from Leopold *et al*. 2006). Discrimination training to various identity morphs leads to responses that increase as the morphs approach the identity of an individual (grey and black thick lines). Thus, the neural code affords some degree of within-category invariance to identity, while maintaining selectivity across identities, even for images near the ‘average’ face. (*f*) In the human medial temporal lobe, cells respond with a remarkable degree of ‘within-category’ invariance for a specific individual. Whereas some cells will prefer only a subset of images of a given individual (thin black lines), many neurons responded to *all examples* of the preferred individual (either left three or right three images), despite large differences in perceptual input, and including the visually dissimilar written name of the individual (thick lines). Bottom images modified from Quiroga *et al*. (2005). (*g*) A relatively unexplored means by which neurons could code for face or object category is in spike timing. In the locust olfactory system, repeated presentation of scent classes evokes fewer, but better-timed responses. (i)–(iii) Subsequent trials of a given odour. (i) The local field potential (population) response, revealing the emergence of sustained oscillations following odour presentation. (ii) The next trace shows the spiking activity, which becomes aligned to the oscillation upon repeated presentations. (iii) Two simultaneously recorded neurons, revealing how time locking to a common oscillation also results in a synchronization of responses across the population of responsive neurons. Fewer, but better-timed spikes may lead to a more efficient representation of the relevant odour classes. Traces are adapted from Stopfer & Laurent (1999).

The most frequently reported change in neural response with perceptual learning appears to be a sparsening of the activity, often seen as a sharpening of a cell's ‘tuning curve’. That is, if a cell responds to several stimuli weakly and to one stimulus strongly, after learning, it might respond only to that ‘best’ stimulus (figure 1*a*-*c*). This sharpening of the response with learning is inferred from the difference in tuning curves of cells responding to trained versus untrained stimuli during discrimination of simple stimulus characteristics such as orientation discrimination (Schoups *et al*. 2001; Yang & Maunsell 2004) or coherent motion from random-dot stimuli (Zohary *et al*. 1994). It has also been described for cells in IT following discrimination training on objects (Baker *et al*. 2002; Sigala & Logothetis 2002; Freedman *et al*. 2003) and can be seen in these cells even after mere exposure to images (Freedman *et al*. 2006).

In principle, sharpening could result from an increase in response to the optimal stimuli, a decrease in the response to sub-optimal stimuli, or a combination of the two. In practice, cells in IT manifest sharpening primarily as a decrease in response to stimuli that elicit a sub-optimal response, rather than an amplification of the best stimulus (Freedman *et al*. 2006), to the point of observing responses below baseline for the least-preferred, trained stimuli.

On the whole, the result of sharpening across the population of cells would lead to a smaller fraction of cells being active for any given stimulus as a result of learning. But the population may also change as a function of learning (figure 1*d*). Discrimination training, particularly in the presence of reinforcement, leads to greater cortical magnification corresponding to the trained locations. That is, more cells respond to the trained location, potentially at the cost of cells responding to nearby locations, which can be depicted as a ‘swelling’ of the cortical map of response preferences. Examples of such cortical reorganization or magnification include expansion of somatosensory areas on two digits that were stimulated in a discrimination task (Recanzone *et al*. 1992*a*,*b*), or in auditory cortex, for regions responding to tones that needed to be discriminated (Recanzone *et al*. 1993). In fact, both sharpening of tuning and a shift in preferred stimuli towards the trained stimuli—as seen in cortical expansion—have been observed (Recanzone *et al*. 1993; Weinberger 1993). In this way, there may not be a loss of net activity for a given stimulus, even with sparsening. Independent of tuning curve changes, an increase in signal (to noise) would result from the addition of cells with selective responses to the stimuli that need to be discriminated.

Changes in the sparseness and coverage—the number of responsive neurons—may cluster around trained or relevant stimuli for discrimination, even in the absence of any obvious cortical ‘map’ or topography. For example, when comparing responses to face and non-face images, more cells responded to faces, but with a more distributed tuning than for non-face-responsive cells (Rolls & Tovee 1995). This led to a greater differentiation of the face stimuli than of the non-face stimuli, consistent with behavioural evidence in humans that individual faces are identified faster and more accurately than within-category object exemplars (e.g. Tanaka 2001). If one presumes greater ‘training’ with faces than non-face objects, this would be one example of a *broader* tuning for the trained set, but leading to greater discriminability of members of the trained set; however, these conclusions are based on only 14 cells, which were located in the fundus of STS. Moreover, faces may constitute a unique category of objects for which innate or early developmental biases may exist (Hoffman & Gauthier 2007), thus their underlying coding scheme may be different from that of other object categories.

### (a) Other possible changes with learning

The above accounts of changes with learning all influence the signal of the rate code in response to stimuli. Another putative mechanism of learning, not mutually exclusive with the others mentioned, involves recruitment of neurons from other brain areas. This could happen though enhancement of the synaptic connections that converge upon downstream areas, by which selectivity could be ‘built up’ for important differences such as the features that define an object or group of objects. Indeed, the prefrontal cortex shows category-boundary sensitivity (Freedman *et al*. 2003), possibly resulting from temporal lobe projections, though the development of this selectivity with learning is unknown.

Change could also occur in upstream areas, as a cascade moving in the reverse direction of signal propagation during stimulus processing. According to this reverse hierarchy theory (Ahissar & Hochstein 2004), the broad spectrum of responses initially passed on to a ‘downstream’ target area narrows according to the features that are necessary for the learned discrimination. If an upstream area contains cells selective for the dimension of discrimination, and if the task consistently activates the same pool of neurons in that area, then a ‘backwards shift’ to that area is predicted. For example, cells in IT do not change tuning for an orientation discrimination task (Vogels & Orban 1994*b*), but their upstream target neurons in V4 do (Yang & Maunsell 2004). Part of the process of perceptual learning, therefore, would be to find the optimal level in the visual hierarchy for maximizing the neural signal to noise available for that task (see Kourtzi & DiCarlo (2006) for additional discussion).

Yet another way of enhancing performance would be to reduce the ‘noise’ in the tuning curves of a given cell through more precise, consistent firing of that cell. When the difference in responses to preferred and non-preferred stimuli can be as little as a few spikes per trial, it may be difficult to determine whether the response on a given trial was a poor response to a preferred stimulus or a strong response to a non-preferred stimulus. Consistent, well-timed responses can be generated when spikes are phase locked to an oscillation. If oscillations are, in turn, evoked in response to a stimulus, reliable spike timing and rate can be realised. In the olfactory systems of locusts, precisely such a mechanism has been observed (figure 1*g*; Stopfer & Laurent 1999). It remains to be seen whether neurons in IT show any timing effects with learning, or if any oscillations develop.

Though inconclusive, it is perhaps of interest that the activation in IT, which is typically analysed as a rate code value, often includes early transient responses. These early responses may be critical for accounts of rapid perception. Monkeys are able to discriminate categories of images by generating the correct manual response around 170–180 ms after stimulus onset (Fabre-Thorpe *et al*. 1998), yet typical onset latencies in IT are approximately 100 ms and rate code values are calculated in windows that end hundreds of milliseconds after these manual responses have occurred. Selective responses to faces (Oram & Perrett 1992) or various objects (Hung *et al*. 2005) can be observed as early as 5–13 ms after the onset of the responses in IT. Often, the initial response includes a transient ‘peak’ and this has been shown to contain information about category membership (Freedman *et al*. 2003). This means that the first 1–2 spikes of a response could provide sufficient information about the stimulus to make perceptual or categorical judgments, consistent with evidence that information may be conveyed in the timing of IT responses (Optican & Richmond 1987; Richmond & Optican 1987; Richmond *et al*. 1987). Oscillatory activity is but one means of accomplishing this, and there is relatively little information about the role oscillations play in IT responses. It is, however, intriguing to note that the population responses in IT to *familiar* objects show approximately 8Hz ‘rebounds’ of activity not seen for the population response to *novel* objects (Freedman *et al*. 2006, fig. 8). In general, though, the role of oscillations or spike timing on the neural basis for object recognition awaits further investigation.

## 3. Object recognition II: generalization and category formation

The mechanisms underlying perceptual learning, and object recognition in particular, must not only hone our ability to discriminate diagnostic or relevant differences between two objects; it must also facilitate recognition of objects as belonging to a single group, despite perceptual differences. Take, for example, the change in the retinal position of a face as we stare at its different parts, and in its size as we approach it; the contrast of a face under different lighting conditions, and the changes in view, from profile to head-on. These variances produce dramatically different images on the retina, yet we perceive the object type in spite of the varied exemplars. This generalization, or invariance, across exemplars is a key component in object learning. We will consider three types of generalization: those to retinal deviations, object view and categorization, operating on the premise that the development of invariances for these parameters occur through experience.

### (a) Invariance to size, position and in-plane rotation

Shape-selective cells in IT show size invariance, meaning that a preferred stimulus will elicit a similar response despite dramatic changes in its size (Sato *et al*. 1980; Desimone *et al*. 1984; Sary *et al*. 1993). These cells also show position invariance, in which centrally presented and increasingly eccentric presentations of the same images elicit similar responses (but see DiCarlo & Maunsell (2003) for limits to position invariance). Since the areas of visual cortex that project to IT are retinotopic, one question was how variations in retinal input, and thus variations in the topographic neural responses, could ultimately be combined to produce invariant responses. Solutions emerged from hierarchical models of translation invariance, which could sequentially build up representations of increasing complexity (Fukushima 1980; Perrett & Oram 1993; Wallis & Rolls 1997). These models typically posit projections from a lower-level retinotopic layer to layers with increasingly large and complex receptive fields. Ultimately, these fields reflecting one location in the visual field send divergent projections to the invariant area. Projections from various retinotopic locations would converge within the invariant area based instead on the ‘match’ to the complexity of the stimulus. But for this type of invariance, all the information needed for the match is present in the input: an image-plane rotation, translation, or scaling ‘aligns’ the stimulus for matching. What about ‘hidden’ parts of three-dimensional objects, which we are able to recognise despite rotations in depth, revealing new features of the image and hiding others?

Object recognition from different views may occur as the result of a transformation of the object until it matches a single stored three-dimensional description of an object, or based on structural descriptions of the key elements of the three-dimensional structure (‘object centred descriptions’: Marr 1982; Biederman 1987; Ullman 1989). Alternatively, view invariance may arise from experience: with a sufficient number of views of an object, novel views may be a close enough match to one of the collection of learned views (‘viewer-centred descriptions’: Poggio & Edelman 1990; Logothetis *et al*. 1994). In practice, both view-based and view-invariant codes have been observed, though the preponderance of view-selective cells strongly suggests viewer-centred descriptions. After extensive training (∼600 000 trials per category) of monkeys on a set of novel three-dimensional objects, a population of cells in IT were revealed to respond to novel views of trained stimuli (Logothetis & Pauls 1995). Of approximately 1000 neurons recorded, the vast majority were visually responsive, and 12 per cent of those were selectively tuned around a preferred view of one preferred object. The tuning varied across the population, such that the object could, in principle, be recognised based on which of the collection of neurons happened to be tuned to the present view. By contrast, only three cells (0.37%) showed view-invariant object preferences. Similar results were obtained by exposing monkeys to real three-dimensional objects in their home cages and then testing neural responses to images of those objects, yielding mainly view-selective but also view-invariant responses (Booth & Rolls 1998). The view-invariant responses would not be necessary for recognition, due to the preponderance of view-tuned cells, thus the overall code appears to fit best with viewer-centre models of object recognition.

### (b) Categorization

At a behavioural level, studies in the pigeon were the first to demonstrate generalization of categories of images such as ‘human’ and ‘non-human’ (Herrnstein & Loveland 1964). In the wild, vervet monkeys naturally ‘categorize’ potential predators, producing unique vocalizations in response (Seyfarth *et al*. 1980). In laboratory settings, monkeys show categorization of images (Humphrey 1974; Schrier *et al*. 1984; Yoshikubo 1985; Schrier & Brady 1987; D'Amato & Van Sant 1988; Roberts & Mazmanian 1988) including the untrained ability to discriminate faces and objects (Pascalis & Bachevalier 1998). Moreover, they show a hierarchical categorization that allows untrained discrimination between their own species' faces and another species of monkey, but also better discrimination between pairs of their own species' faces than between pairs of the other species' faces (i.e. species-specific subordinate-level discrimination; Dahl *et al*. 2007). What neural activity underlies these abilities?

Invariance could be manifest in cells that fire for all exemplars of one category and none of any others. But other possibilities exist. Invariance could also arise from a population of category-specific neurons that still maintain selectivity for some examples within the category. For example, basic-level categories such as trees and fish (Vogels 1999), or trained categories of image morphs from virtual ‘cat’ and ‘dog’ stimuli (Freedman *et al*. 2003), evoke responses in IT. These responses discriminated categories, not by showing similar responses across all members within a category, but by showing some preference for many category members. In fact, cells with broad tuning (a distributed code) but favouring one category over another produced the best decoding of categories (Thomas *et al*. 2001). Thus, it is from the neural population activity, and not necessarily exclusive ‘category cells’, that category membership can be extracted.

Sometimes the shape space among parametrically varied stimuli appears to be the main coding dimension. Op de Beeck *et al*. (2001) did not find cells that differentiated between two categories, nor was there any ‘boundary’ effect by which selectivity was the greatest near category borders; rather, the activity they recorded reflected the differences between their parametrically varying radial-basis function stimuli, irrespective of category membership. Perhaps this was due to the low dimensionality of the stimuli—or the training on different spatial arrangements of category boundaries across the three sets of stimuli.

Another set of systematically varied stimuli with considerably more complexity is the ‘face space’ created from morphs of multiple three-dimensionally rendered faces. Where identity may be represented as distance from a core prototype, one might expect cells that are sensitive to deviations from the prototype, allowing effective grouping among exemplars of one identity, but discrimination among exemplars across identity lines. Indeed, in monkeys that had seen many examples of morphed faces that varied in their proximity to a face prototype, activity of a given cell tended to increase with increasing distance from the prototype, for a subset of identities (figure 1*e*; Leopold *et al*. 2006). This monotonic increase in preference would help differentiate among various exemplars that fit in face space around a prototype (Loffler *et al*. 2005). In addition, it suggests an emphasis on the representation of the distinguishing characteristics in a set of stimuli, with some invariance towards changes that are not diagnostic for identity in face space (i.e. radial changes). It should also be noted that this face space, though not explicitly reinforced in one of the monkeys studied, was viewed repeatedly hundreds of times over many daily sessions. It is not unreasonable to suppose that there might be an adaptive coding to ‘fill’ the face space in terms of neural selectivity. Thus, it is not clear if prototype coding is a default scheme adopted by IT neurons, if it is merely a consequence of efficiently differentiating this regular, predictable face space, or if faces constitute a special class of objects with unique coding properties. In any case, these results highlight an important balance in the process of categorization between sensitivity and generalization.

Of all the features that differ across objects, categorization requires that features differing between within-category members be ignored, and features that are similar within-category but that are not shared by members of other categories should be detected. Indeed, there is evidence that the ‘diagnostic features’ of a category—those that are most telling of category membership—are preferentially encoded in IT neurons (Sigala *et al*. 2002; Nielsen *et al*. 2006). This places an emphasis on the functional differences among exemplars, and not necessarily their physical similarity. And this emphasis may be one of the key features of how the brain categorizes objects.

### (c) Processing amodally

Amodal categorization is the grouping of common stimuli independent of the modality of sensory input. Primates show behavioural signs of amodal categorization/cross-modal equivalence. When provided with an object to inspect haptically, apes and monkeys were able to generalize what they had learned to the visual modality (Davenport & Rogers 1970; Weiskrantz & Cowey 1975; Elliot 1977). In addition, when monkeys (or humans) were expected to categorize vocalizations, prior presentation of conceptually congruent images led to faster responses (Martin-Malivel & Fagot 2001). This demonstrates an independence of the perceptual attributes of a stimulus to categorization, something that should be incorporated more explicitly in the aforementioned models of object categorization.

We do not yet know the neural basis for amodal processing, and, indeed, there may be biases for some stimulus pairings across modality that are innate or biased early in development, and therefore are not consistent with other types of category learning. Nevertheless, associations have been described for arbitrary (but rewarded) pairings of stimuli, suggesting cross-modal or amodal representations can be learned under the same conditions as other types of categorization. There are hints that IT and PRh may accomplish this by coding for associated stimuli, irrespective of their featural similarity. After monkeys have learned the arbitrary pairing of sets of images, ‘pair coding’ neurons—those responding selectively to both images in a pair—are seen (Miyashita 1988; Sakai & Miyashita 1991). The paired associations in IT neurons parallel learning (Messinger *et al*. 2001) and can develop even without explicit reinforcement, that is when the second item in the pair is irrelevant to correct task performance (Erickson & Desimone 1999). Other associations have been reported in monkeys well trained to associate one visual cue with one auditory cue, and a second visual cue with a second auditory cue (Colombo & Gross 1994; Gibson & Maunsell 1997). Under these conditions, cells were found in ‘visual’ cortex which were selective for one of the trained auditory cues, suggesting a powerful association that could also underlie amodal categorization.

In humans, neurons in the medial temporal lobe have shown a striking degree of invariance, in which the written name of a preferred individual was sufficient to drive the cell as strongly as the response to various images of that individual, whereas other visually similar individuals were ineffective stimuli (figure 1*f*; Quiroga *et al*. 2005). That degree of invariance has not yet been described in the monkey, but this could be due to several differences between many object–response studies in monkeys and the human study. First, the hippocampus and entorhinal cortex were the regions demonstrating invariant responses to individuals. The IT was not tested in humans, and typically, the hippocampal regions are not tested for these properties in monkeys. Second, the spontaneous firing rates of the invariant cells were often remarkably low; well under 1 Hz. Biased selection of IT cells during electrode placement would likely lead to higher spontaneously active neurons, or even neurons explicitly active for a broad range of stimuli, thus sparse-firing cells such as the invariant neurons may have been passed by in the monkey and in previous human recordings. Finally, the cell isolation procedures have improved greatly, and sorting of spikes requires good signal-to-noise ratio on the electrode as well as proper isolation procedures. Many of the studies from IT used straight threshold crossing or pre-determined spike waveform templates that could have excluded the very cells that demonstrate invariance, or possibly included them as part of a multiple unit activity dominated by higher firing rate cells. Further testing using procedures designed for unbiased sampling and optimal cell isolation, in addition to recording in both lateral and medial temporal lobe regions, could resolve this outstanding issue.

The learning-induced changes in object-selective neurons show some similarities to neurons in other visual areas following perceptual learning. Namely, neural activity is recruited to code the stimuli or features to be differentiated, and a given neuron's activity will be more sharply tuned around the diagnostic parameters. Broadening of selectivity has also been observed, so determining when a distributed code will emerge and when sharpening or sparsening follows learning will be a matter for future studies. Category selectivity also has been seen in these cells, even concurrent with increases in sensitivity. That is, in IT, category membership can be extracted from the tuning of category-biased cells, rather than appearing in dedicated ‘within-category invariant’ responses. This is in contrast to a small group of cells recorded in the human medial temporal lobe, which, to a large extent, do seem to reflect within-category invariant responses around each example of a given individual. In addition, the prefrontal cortex in the monkey shows a greater degree of within-category invariant responses. Where IT cells do approach a remarkable degree of invariance is in the arbitrary pairing of stimuli that become associated with each other. This suggests that the temporal lobe is capable of associating and generalizing, provided there is a strong basis of experience on which to base those associations. Amodal associations would, therefore, be an interesting domain to pursue in the monkey, in particular for stimuli with which the monkey has an abundance of experience. Another important task will be to determine whether the discrepancy between invariances to the individual in humans, and its relative absence in the monkey temporal lobe, is a consequence of brain region, species or some other as-yet-unknown factor. In sum, the IT undergoes a wide range of changes with object learning, supporting a delicate balance between sensitivity to relevant differences, generalization across irrelevant differences and the ability to flexibly adapt to both.
